# The Development of Practice Recommendations for Drug-Disease Interactions by Literature Review and Expert Opinion

**DOI:** 10.3389/fphar.2020.00707

**Published:** 2020-05-15

**Authors:** Justine M. Z. van Tongeren, S. Froukje Harkes-Idzinga, Heleen van der Sijs, Roya Atiqi, Bart J. F. van den Bemt, L. Willem Draijer, Deline Hiel, Adrian Kerremans, Bart Kremers, Marc de Leeuw, Marleen V. Olthoff, T. Kim-Loan Pham, Ricky Valentijn-Robertz, Kayan Tsoi, Iris Wichers, Maaike de Wit, Sander D. Borgsteede

**Affiliations:** ^1^ Department of Clinical Decision Support, Health Base Foundation, Houten, Netherlands; ^2^ Medicines Information Centre, Royal Dutch Pharmacists Association (KNMP), The Hague, Netherlands; ^3^ Department of Hospital Pharmacy, Erasmus University Medical Center, Rotterdam, Netherlands; ^4^ Department of Internal Medicine, University Medical Center Groningen, Groningen, Netherlands; ^5^ Department of Pharmacy, Sint Maartenskliniek, Nijmegen, Netherlands; ^6^ Department of Pharmacy, Radboud University Medical Center, Nijmegen, Netherlands; ^7^ Huisartsenpraktijk Bij de Haven, Nijkerk, Netherlands; ^8^ Department of Hospital Pharmacy, Alrijne Zorggroep, Leiden, Netherlands; ^9^ Independent Researcher, Helmond, Netherlands; ^10^ Ravenstein apotheek, Ravenstein, Netherlands; ^11^ De Vijverapotheek, Nieuwkoop, Netherlands; ^12^ Department of Guideline Development and Research, Dutch College of General Practitioners, Utrecht, Netherlands

**Keywords:** drug-disease interactions, literature review, expert opinion, study protocol, practice recommendations, clinical decision support

## Abstract

**Background:**

Drug-disease interactions negatively affect the benefit/risk ratio of drugs for specific populations. In these conditions drugs should be avoided, adjusted, or accompanied by extra monitoring. The motivation for many drug-disease interactions in the Summary of Product Characteristics (SmPC) is sometimes insufficiently supported by (accessible) evidence. As a consequence the translation of SmPC to clinical practice may lead to non-specific recommendations. For the translation of this information to the real world, it is necessary to evaluate the available knowledge about drug-disease interactions, and to formulate specific recommendations for prescribers and pharmacists. The aim of this paper is to describe a standardized method how to develop practice recommendations for drug-disease interactions by literature review and expert opinion.

**Methods:**

The development of recommendations for drug-disease interactions will follow a six-step plan involving a multidisciplinary expert panel (1). The scope of the drug-disease interaction will be specified by defining the disease and by describing relevant effects of this drug-disease interaction. Drugs possibly involved in this drug-disease interaction are selected by checking the official product information, literature, and expert opinion (2). Evidence will be collected from the official product information, guidelines, handbooks, and primary literature (3). Study characteristics and outcomes will be evaluated and presented in standardized reports, including preliminary conclusions on the clinical relevance and practice recommendations (4). The multidisciplinary expert panel will discuss the reports and will either adopt or adjust the conclusions (5). Practice recommendations will be integrated in clinical decision support systems and published (6). The results of the evaluated drug-disease interactions will remain up-to-date by screening new risk information, periodic literature review, and (re)assessments initiated by health care providers.

**Actionable Recommendations:**

The practice recommendations will result in advices for specific DDSI. The content and considerations of these DDSIs will be published and implemented in all Clinical Decision Support Systems in the Netherlands.

**Discussion:**

The recommendations result in professional guidance in the context of individual patient care. The professional will be supported in the decision making in concerning pharmacotherapy for the treatment of a medical problem, and the clinical risks of the proposed medication in combination with specific diseases.

## Introduction

Contra-indications are situations where a medical product must not be given for safety reasons (European Commission; U.S. Department of Health and Human Services Food and Drug Administration) Depending on the patient characteristics, there may be different reasons for contra-indications, such as physiological conditions (age, gender, pregnancy, etc), hypersensitivity or a concomitant disease (European Commission; U.S. Department of Health and Human Services Food and Drug Administration). Drug-disease interactions (DDSIs) are situations where the pharmacotherapy used to treat a disease causes worsening of another disease in a patient (Merck Manual Consumer Version). In these circumstances drugs should be avoided (i.e., are contra-indicated), adjusted or accompanied by extra monitoring. DDSIs are common; a recent study showed that 13.9% of all prescriptions in community pharmacy generated a drug therapy alert regarding a DDSI (Heringa et al., 2016). In elderly patients, 15%–16% of the patients had at least one drug-disease interaction (Lindblad et al., 2006; Hanlon et al., 2017). Managing of DDSI is recommended to prevent serious harm or death (Shastay, 2017).

The official product label is an important source of information regarding DDSIs. The European Summary of Product Characteristics (SmPC) and the U.S. FDA label information describe (in section 4.3 and section 4, respectively), contra-indications (European Commission; U.S. Department of Health and Human Services Food and Drug Administration) However, the official product label does not take into account that this could leave patients without treatment, since alternatives may not be available, have proven to be ineffective or are also contraindicated. This leaves the question whether it is best to avoid potential risks of contraindicated drugs and leave the disease untreated or to accept the risks to maintain the possibility of treatment. Another problem regarding DDSIs occurs to the SmPC section 4.4 “Special warnings and precautions for use”, and the FDA-label section 5 “Warnings and precautions” (European Commission; U.S. Department of Health and Human Services Food and Drug Administration). These sections describe specific risks that should lead to a precaution for use or informing healthcare providers. The official product information does not always provide information on how these risks affect these specific patient groups. In a recent study, Weersink et al. showed that the clinical applicability of information in the SmPC for drug use in cirrhosis was problematic due to ambiguously formulated information (Weersink et al., 2019). Therefore, it is unclear whether the drug should be substituted, if the dosage should be changed, or if additional information or monitoring is required. A complication of the SmPC is that it is also a document with a legal status, and has to meet the requirements that must be met for the inclusion of warnings and prohibitions.

Patients with comorbidities are often excluded from clinical trials, which limits the generation of evidence regarding safety of drugs in specific patient groups (Van Spall et al., 2007; Boyd et al., 2012; Duma et al., 2019). Even when potential risks are detected, it is difficult to prove causality due to a low incidence of negative outcomes related to DDSIs. Information on DDSIs is also relatively uncommon in treatment guidelines (Dumbreck et al., 2015). For some DDSIs however, practice guidelines are available for specific patient populations with respect to patient characteristics and/or pharmacokinetics, e.g., pregnancy/lactation (Addis et al., 2000), porphyria (Thunell et al., 2007), long QT syndrome (Woosley et al.), renal impairment (Ashley and Dunleavy, 2018; Royal Dutch Pharmacists Association, 2019), and liver cirrhosis (Weersink et al., 2016). For other relevant diseases, it is likewise desirable to manage DDSIs to optimize both disease management and pharmacotherapy, and to minimize harm. These recommendations should guide the health care professional with respect to the clinical relevance of the DDSI as well as the possible actions that can be considered. It is essential that these recommendations are based on standardized, transparent reports that summarize the available evidence of information from the SmPCs, scientific literature and expert opinions, and translate this into advices that are applicable in clinical practice (Floor-Schreudering et al., 2014).

Since the 80’s, alerts for DDSIs have been introduced in Clinical Decision Support systems in the Netherlands (De Gier, 1986; De Smet, 1988). The evaluation of potentially clinical relevant DDSIs has evolved from different opinions of individuals and working groups in the Netherlands to the current national protocol that combines literature review and expert opinion. The aim of this paper is to describe the systematic method to develop practice recommendations for drug-disease interactions.

## Methods

**Figure 1:**
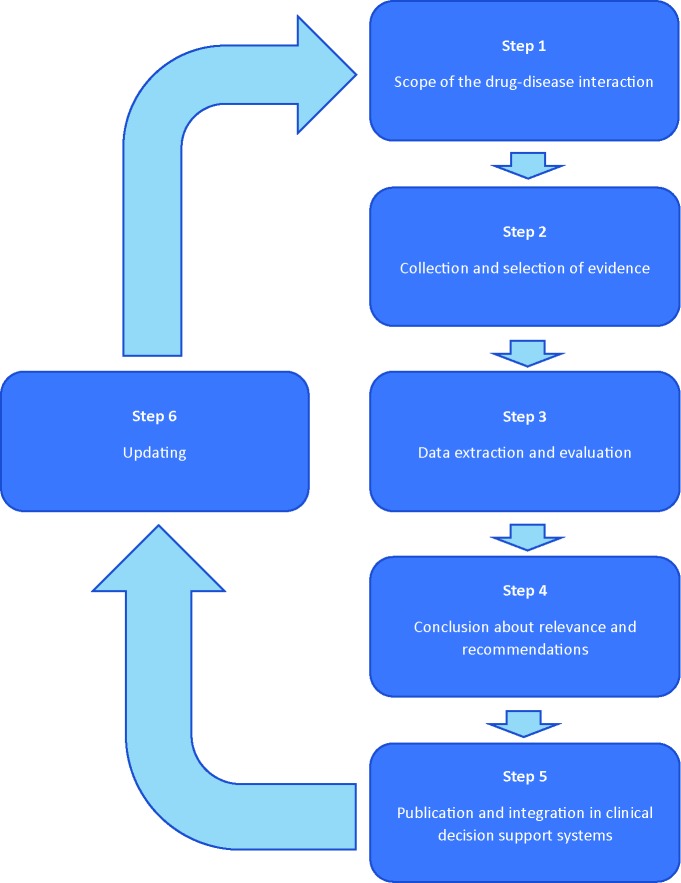
Flow chart of the evaluation plan.

Practice recommendations of DDSIs will be assessed following a six step plan, forming a continuous circle of evaluation (**Figure 1**). The assessment will be performed by two pharmacist-reviewers: experts in evaluation of the safety of drugs in the context of clinical decision support. Both reviewers are trained in literature review according to Cochrane methods for literature extraction and quality assessment. A multidisciplinary expert panel will be involved in step 1 and 4. The expert panel will include a fixed panel with twelve health care professionals of the following disciplines: (1) specialists who have experience in prescribing a wide range of drugs (2 general practitioners, 1 internist/clinical pharmacologist); (2) pharmacists in primary and clinical care (2 community pharmacists, 1 community pharmacist/clinical pharmacologist, and 1 hospital pharmacists, 1 hospital pharmacist/clinical pharmacologist); (3) specialists involved in professional organisations with respect to guidelines for pharmacotherapy or clinical decision support (n=4). Additionally, there are rotating chairs available for additional expertise, such as physicians from the medical (sub)specialties to be involved for specific DDSI assessments. For example, a neurologist and psychiatrist will be added to the expert panel to include their perspectives when evaluating a DDSI between anti-epileptics and psychotic disorders. The number of additional chairs will vary between specific drug-disease interactions. Consensus will be used to achieve conclusions. Potential conflicts of interest of the expert panel are identified and published.

The role of the expert panel will be (A) to determine the scope of the DDSI, and (B) to conclude about the clinical relevance and recommendations (see step 1 and step 4 for detailed information).

### Step 1: Scope of the DDSI

#### Scope of Disease

For every DDSI, three aspects should be identified:

1. Definition of Disease.
  Internationally accepted classification systems such as International Classification of Primary care (ICPC) (De Smet, 1988) or International Statistical Classification of Diseases and Related Health Problems (ICD) (WONCA) are preferably used to specify a disease of state. For example, a delirium and psychosis have an overlap in clinical presentation but have a different etiology. The expert panel should decide and motivate if in case of the assessment of a DDSI regarding drug X and disease psychotic disorder, data on delirium, and/or psychosis should be included. Although diseases in literature are not always classified using such classification systems, it is important to specify which diseases are included.
2. Definition of Clinically Relevant Potential Effects.
  A disease can worsen in various degrees. To maintain a clear line in the assessment process of all DDSIs regarding disease Y, the minimal level of worsening regarded as clinically relevant should be defined. This clinical relevance is the impact of the drug on the worsening of disease, and is specified by (1) the impact on clinical outcomes and/or the quality of life, and (2) the expected odds that the worsening will happen. A rise in systolic blood pressure of 1 mmHg is not regarded clinically relevant in patients with hypertension. However, any seizure in previously seizure-free epilepsy patients is generally regarded clinically relevant because of the impact on patients’ lives.
3. Relevance of Treatment.

It needs to be determined whether the risk of a DDSI persists after successful treatment (hypertension under control) or cure (mamma carcinoma).

#### Selection of Drugs to be Evaluated

Drugs to be evaluated will be selected according to the following sources: (1) information in the official product information (SmPC), (2) published information in medical literature, and (3) handbooks and professional guidelines.

1. SmPCs: All SmPCs from products marketed in the Netherlands will be explored. The sections “contraindications” (4.3) and “special warnings and special precautions for use” (4.4) will be searched for drugs to be selected. When a disease is named in one of these sections, the DDSI will be evaluated.
2. Medical Literature: for each disease a literature search will be performed in electronic literature databases (Pubmed, EMBASE) to identify potential drugs that may be involved in a DDSI based on either evidence or pharmacological profile. Reviews about the pathophysiology and pharmacotherapy in specific diseases will be selected for this purpose.
3. Handbooks and professional guidelines will be searched to identify potential drugs involved in DDSIs.

The expert panel will evaluate the drugs found in step 1–3 and will add or remove potentially relevant drugs based on motivated expert opinion.

In conclusion, at the end of step 1, the disease and the drugs to be evaluated will be defined. This will be the starting point of the evaluation of the specific drug-disease combinations.

### Step 2: Collection and Selection of Evidence

For each drug, evidence will be collected with respect to the risks of its use in the context of the disease Y. The evidence will be clustered together for drugs that are expected to cause similar worsening of the disease through shared pharmacological properties based on similarities in the chemical structure and receptor binding profile, a common pharmacological mechanism of action and side effects profile (e.g., dihydropyridine calcium channel blockers and antihistamines). Potential risks for the use of drug X in disease Y can be based on the findings presented in **Table 1**.

**Table 1:** Findings suggestive of a drug-disease interaction.

Finding	Example
Drug X causes worsening of disease Y	Aggravation of psoriasis by beta-blockers
Drug X causes symptoms related to disease Y	Mania symptoms caused by corticosteroids
Drug X causes an increased risk to develop disease Y	Higher frequency of ischemic strokes in patients using combined oral contraceptives
A theoretical relation exists between the pharmacological characteristics of drug X and the pathophysiology of disease Y	Potentially harmful stimulation of dopamine receptor D2 by dopamine agonists in schizophrenia.

#### Collection of Evidence

Information will be collected in the following sources:

- *SmPC and Public Assessment Report (PAR):* The following sections in the SmPC will be searched for relevant information about the DDSI: “Posology and method of administration” (4.2), “contraindications” (4.3), “special warnings and special precautions for use” (4.4), “side effects” (4.8), “pharmacodynamics properties” (5.1), and “pharmacokinetic properties” (5.2). Whenever available, the PAR will be searched for additional information. The registration holder will be contacted for more information if data in the SmPC or PAR are unclear.
- *Guidelines and Handbooks:* Handbooks and (inter)national guidelines about the specific disease will be searched for information regarding the DDSI.
- *Electronic Databases:* A literature search will be performed in electronic databases (Pubmed, EMBASE) using keywords for the following search items: disease, drug name/drug class, and terms that identify risks associated with the combination (e.g., avoid, risk, adverse drug reaction). In case of diseases with very different clinical presentations, terms that will be used for identifying the population will be aligned with the expert panel at the end of step 1. Subheadings, Booleans, and title words will be used to refine the strategy. Additional publications will be added through citation tracking.

##### Exclusion Criteria

**Figure 2:**
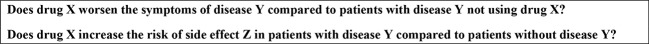
Key questions in drug-disease interactions.

When a drug is contraindicated for reasons of ineffectiveness or the availability of better treatment options, this will not be regarded as evidence supporting a DDSI. This will be motivated in the assessment report. Other exclusion criteria are studies that do not involve humans and publications lacking information on the key questions in **Figure 2**.

#### Selection of Evidence

The following criteria will be used to select the evidence:

1. Contribution to answer the research question. Generally, few studies have been performed in the population with the disease in question. All available publications concerning the population at risk will be included. If not available, publications regarding drug X causing disease Y will be included. A specific type of papers are those describing the potential pharmacological mechanism. These papers might contribute to answering the research question, although they often do not include patient data.
2. Level of evidence. We will include the publications of clinical information with the highest level of evidence (meta-analysis, systematic reviews, clinical trials, and other controlled studies). Other sources, such as case reports, will be included to underline the potential clinical relevance. The quality of the evidence concerning the population at risk was evaluated by grade 1 to 4 based on established criteria for drug-drug interactions that were used in previous studies (Van Roon et al., 2005), slightly adjusted for DDSIs (**Table 2**). Other studies and data were evaluated quantitatively.

The exact search strategy and criteria for the selection of literature will be motivated in every DDSI report. **Table 2**.

**Table 2:** Quality of Evidence for drug-disease interactions (DDSIs).

Definition	Score
Meta-analyses, systematic reviews, randomized controlled trials with clinically relevant endpoints	4
Controlled trials in patients with surrogate endpoints; observational studies	3
Well documented case reports; case series	2
Incomplete published case reports (no re- or de-challenge, presence of other explaining factors for the adverse reaction)	1

### Step 3: Data Extraction and Evaluation

#### Extraction

For every DDSI assessment, summary tables will describe the following characteristics and results of the included information: study type, number of patients with and without disease Y, drug regimen, outcomes, and level of evidence of the study. The outcomes will describe the identified risks, including severity of the disease and statistics if present. Additional risk factors are presented quantitative whenever available.

Summary tables will also present the relevant data originating from the SmPC and relevant data from handbooks and or guidelines.

#### Evaluation

The data obtained in step 3 will be presented as an assessment report with motivated answers to the following questions: (a) is there a clinically relevant DDSI, and (b) is an alert required in clinical decision support systems.

- The data will be considered in the context of the patients at risk for a clinically relevant interaction between drug X and disease Y (Yes/No) using the criteria determined in the scope of the DDSI (see step 1). When evidence for specific formulations is lacking, pharmacokinetic data, and data on systemic effects will be reviewed and used to decide whether extrapolation is justifiable. In all the reports, the reviewers will evaluate if the additional risks can be attributed to the individual drug or the entire drug class. The evaluation will be based on literature and pharmacologic characteristics of the drug(s). A preliminary conclusion about the DDSI will be formulated.
- When sufficient evidence of a clinically relevant interaction between drug X and disease Y is available, another question will be answered: does the DDSI require an alert in the clinical decision support systems (Yes/No)?

Alerts are often overridden which is partly caused by their appearance in situations without peril or not requiring action (Van der Sijs et al., 2006; Isaac et al., 2009; Nanji et al., 2014). Alerts can be irrelevant when a risk has already been weighed against the benefits by the prescriber. This situation may occur when (1) appropriate guidelines have been established and are implemented, (2) the need for treatment outweighs the potential risk and the risk is properly monitored, and/or (3) when both drug X and disease Y are prescribed and treated by a specialized discipline. For example, when there is a DDSI between a cardiovascular drug and a cardiovascular disease, an alert may not be needed when the drug is only prescribed by cardiologists, the effect is mild, and there are no alternatives available. In these situations, the assessment report motivates the reason why the DDSI does not require a drug alert for clinical decision support.

When an alert is required, an alert message will be proposed with information about the clinical risk, risk factors and decision support. Risk factors are factors that are associated with a higher probability of the occurrence of the clinical risk. The decision support consists of four types of advice that will be composed for the specific DDSI (**Table 3**). Multiple advices can exist for the same drug, depending on the indication, prescriber, and context, since the benefit/risk ratio may differ. Decision support advices (**Tables 3**:2–4) may include dose adjustments. **Table 4** summarizes factors considered for deciding on a drug alert and if deemed necessary, the content of the advice. The content and design of the extraction forms were piloted and reviewed for feasibility by the expert panel.

**Table 3:** Types of decision support advices and factors considered for assigning an advice to a Drug-disease interaction (DDSI).

Avoid drug X in disease Y. Avoid drug X unless carefully considered by the specialist treating disease Y. Monitor DDSI effect.^#^ Avoid drug X, unless there is no alternative available. In that case, monitor DDSI effect.^#^ Monitor DDSI effect.^#^

^#^Dose adjustment optional.

**Table 4:** Factors considered for assigning an advice.

- Availability of alternatives for drug X - Severity of effect DDSI - Relation between the disciplines involved in prescribing drug X and disciplines treating disease Y - Ability to monitor the DDSI effect - Risk factors

### Step 4: Conclusion About Relevance and Recommendations

The expert panel is provided with all used information and decides on the literature selection criteria (step 2), summary, and conclusions of the literature, the proposed categorization and contents of the advice (step 3). The reports will be supplemented with expert opinions generated in the discussions. These may also involve evaluation of current handling of the (presumed) DDSI and practical implications of the proposed handling; every discipline should be able to practice the decision support advice. If there are different opinions within the expert panel, these will be included as comments in the assessment report. The expert panel will conclude by consensus. The conclusion will be included in the reports.

### Step 5: Publication and Integration in Clinical Decision Support Systems

The assessment reports will be published in Dutch guidelines for DDSIs (Borgsteede et al., ; Royal Dutch Pharmacists Association, 2019). The advices will be integrated in the national drug databases G-Standaard and PharmabasePro in The Netherlands. Hence, they will be adopted in all Dutch Healthcare Information Systems supporting prescribing and dispensing processes in hospitals as well as primary care. Whenever drug X will be prescribed/dispensed to a patient labelled with disease Y, an alert will be generated automatically when deemed necessary as decided in step 3 and 4. Relevant background information concerning the advices will be accessible from the clinical decision support system and available on the websites of the guidelines for DDSIs and will include a reference to the assessment report. The format of an assessment report is included in **Appendix 1** in **Supplementary Material**

### Step 6: Updating

Three main pathways are identified for keeping the assessment reports, related advices, and background information up-to-date:

1. New drugs and new risk information on existing drugs will be screened to identify eligible drugs to be submitted in step 1 of the assessment procedure. New risk information is defined as a news item from the regulatory authorities (Dutch Medicines Evaluation Board, European Medicines Agency).
2. Periodically, all DDSIs for disease Y will be reviewed from step 1. When the results of step 1 have changed, new assessment reports may have to be written. When the results of step 1 have remained the same, the existing reports will only be updated with new literature published after the previous search. In this case, step 3 and 4 are more focussed on whether the earlier made decisions are affected by new evidence or changes in medical practice (e.g., patients monitoring their own blood pressure instead of monitoring by the doctor). For every new drug a new assessment report will be written.
3. Health care providers can also initiate a (re)assessment. This situation may particularly occur when medical practice has changed.

### Ethics

As there were no patients nor data directly derived from patients involved in our methodology, this protocol was not evaluated by an ethics committee. Also, there were no privacy issues with respect to study data applicable.

### Implications

Not all drug-disease combinations will be associated with clinical questions about safety of medications and dose recommendations. To develop the relevant recommendations for DDSI, we will need input from clinical practice for drug-disease combinations they experience clinical problems. Moreover, a multidisciplinary expert panel needs to be initiated to develop recommendations. Finally, drug databases need to implement this knowledge in their database and be able to transfer this to Clinical Decision Support Systems in hospitals and primary care.

## Actionable Recommendations

We expect that the development of practice recommendations will result in advices for specific DDSI. The content and considerations of these DDSIs will be published in national guidelines, professional journals, and international scientific journals. To enhance the application in clinical practice, the DDSIs will be implemented in all Clinical Decision Support Systems in the Netherlands.

### Anticipated Results

A summary will be made to present the findings of all drugs evaluated in the context of a DDSI. This summary will consist of the description of the disease with in- and exclusion criteria, and the substance names of medications that have been evaluated for potentially clinical DDSI, with a conclusion and motivation if the DDSI is considered relevant (see **Table 5**). The summary will also present recommendations how to act if these medications are prescribed, e.g., if the combination can be used safely, if dose adjustments are necessary and if monitoring of the patient is required. The motivation includes pharmacological considerations and experts opinions. All results will be published in national guidelines. Recommendations that need specific professional attention will be published in national professional journals. If recommendations will give a significant contribution to international perspectives of medication safety and translation of knowledge to clinical practice, we aim to publish in international journals as well.

**Table 5:** Summary of findings for a drug-disease interaction.

Disease				
Date conclusion expert panel: *dd/mm/yyyy*				
*Description of disease with in- and exclusion criteria*				
Drugs evaluated				
Drug name (generic)	DDSI (yes/no)	Drug alert required (y/n)	Motivation	Practice recommendation
*Drug (class) 1*				
*Drug (class) 2*				
*….*				

### Implementation in Clinical Decision Support

Finally, the recommendations for clinical decision support will be implemented in the two National Drug Databases in the Netherlands (Pharmabase and G-Standaard). As information of these databases is used in all Health Care Information System in primary care and hospitals, this approach will guarantee a nationwide implementation of the developed DDSIs.

## Discussion

We have developed a systematic method to develop practice recommendations for DDSIs. This method will provide standardized reports of combined evidence and expert opinions about DDSIs, that can be adapted to the specific demands and criteria that might differ between diseases. This protocol facilitates transparent communication about the scientific and clinical motivations of potential DDSIs, and their relevance for decision support in patient care.

The strengths of this method are the combination of evidence and expert opinion, the multidisciplinary character of the advices and the integration in clinical decision support systems. First, there is often limited evidence available in DDSIs, which makes it more important to rely on expert opinions; the clinical and pharmacological experience complement the evidence. Second, the multidisciplinary character of the expert panel ensures that the proposed advices include the available knowledge and experience, and are applicable for all disciplines. Third, the specific actions suggested by alerts of decision support systems enforces implementation in daily practice. These advices are integrated in all Dutch clinical decision support systems for prescribers and pharmacists, in primary and secondary care. This can be regarded as a limitation, since international dissemination is desirable. To support international dissemination, we have published our study protocol and will publish the results of DDSI assessments in international scientific journals as a first step to overcome this limitation. Another limitation of this method is, that it is more time consuming compared to individual opinions, or a single literature source such as the SmPC. However, this effort will result in DDSIs that are evidence and expert-opinion based, and hence we believe the recommendations will be better accepted in clinical care.

However, these assessments cannot replace the information in the SmPC. Where the SmPC should provide information on all potential DDSI, a specific evaluation should reveal the clinical relevance of and conclude about the meaning for clinical practice. Integration of all possible risks regarding DDSIs in clinical decision support systems will lead to a drug alert overload. Important drug alerts may be accidentally overridden, a consequence that is known as “alert fatigue” (Van der Sijs et al., 2006; Heringa et al., 2016). By discussing the risk of alert fatigue in advance, as this method proposes, the benefit of an alert in terms of contribution to drug safety to the risk of alert fatigue is carefully weighed and therefore increases the specificity of drug alerts. This method is complementary to other proposed strategies for reducing alert fatigue, such as individualization of drugs alerts by incorporating more patient characteristics (Van der Sijs et al., 2006; Heringa et al., 2016), using other triggers (e.g., new laboratory values, worsening of disease) (Van der Sijs et al., 2006; Heringa et al., 2016) and clustering signals consisting of the same advices (Heringa et al., 2017).

A previous study has shown that in one-third of patients one or more DDSI alerts were not generated due to missing data in pharmacy practice (Floor-Schreudering et al., 2013). Physicians, pharmacists and patients should co-operate to identify missing drugs and diseases in electronic health records. By allowing patients and health care providers to have access to and suggest changes in their electronic health records, the problem of missing data can be overcome. This enables the handling of DDSIs as proposed by the assessments resulting from our protocol.

The assessments resulting from our protocol will produce advices for health care providers, albeit patients should also be involved in the handling of DDSIs; DDSIs should always be considered in the context of the individual patient, and these kind of decisions should ideally be the result of shared decision making (Oshima Lee and Emanuel, 2013). Patients are always affected by the handling of DDSIs, through the impact of changes in the proposed drug regimen, extra monitoring by their physician (e.g., more consults, blood tests), and/or worsening of symptoms. They also have a prominent role in monitoring their own symptoms and contacting the health care provider when the disease deteriorates. Proper informing of patients is therefore essential. Although there are concerns that negative expectations may cause adverse effects and decrease the efficacy of a drug (the “nocebo effect”) (Bingel, 2014), a recent systematic review pointed out that there is currently not enough evidence for a negative impact of informing patients about side effects (Jose and AlHajri, 2018). It is however important to consider factors that may affect the understanding of information and the related expected outcomes, such as the format (e.g., multimedia approaches) and health literacy (Peters et al., 2011; Jose and AlHajri, 2018).

Methods have been designed for determining the clinical relevance of drugs risks, such as drug-drug interactions (Van Roon et al., 2005) and anticancer drug interactions (Jansman et al., 2011). This protocol matches these existing methods and therefore complements the scientific basis of clinical decision support.

Evaluated DDSIs will lead to scientific publications about these DDSIs using this protocol, and knowledge integrated in decision support followed by interventions that will eventually improve drug management. Future research should focus on (a) closing the current knowledge gaps, (b) the degree of compliance and reasons to differ from the advices, and (c) the patient outcomes.

In conclusion, the procedure of a structured evaluation of DDSIs is described. The advices will generate clinical decision support for healthcare professionals based on available evidence and expert opinion, which enables optimal patient care. Finally, this will also identify gaps in existing knowledge in DDSIs that will form a research agenda for future investigations.

## Author Contributions

JT and SB drafted the manuscript. JT, SH-I, HS, RA, BB, LD, DH, AK, BK, ML, MO, TP, RV-R, KT, IW, MW, and SB contributed to the protocol design. JT, SH, HS, BB, LD, AK, MO, TP, IW, and SB critically revised the manuscript. All authors read and approved the final manuscript.

## Conflict of Interest

The authors declare that the research was conducted in the absence of any commercial or financial relationships that could be construed as a potential conflict of interest.

## Abbreviations

DDSI, Drug-disease interaction; FDA, Food and Drug Administration; ICD, International Statistical Classification of Diseases and Related Health Problems; ICPC, International Classification of Primary Care; PAR, Public Assessment Report; SmPC, Summary of Product Characteristics.
