# Supplementary material for: The Development of Practice Recommendations for Drug-Disease Interactions by Literature Review and Expert Opinion
Source: Front Pharmacol. 2020 May 15;11:707. doi: 10.3389/fphar.2020.00707 (PMC7243438; doi:10.3389/fphar.2020.00707)
Supplement: Supplementary file 1 [file DataSheet_1.pdf]

# Appendix 1 – Format of an assessment report

| ***Disease – drug****<br>*Date literature search* | | | |
|---|---|---|---|
| **Abbreviations** | | | |
| *Abbreviation* | | *Full wording* | |
| **Discussion** | | | |
| *Summary most important findings*<br>*Considerations expert panel* | | | |
| **Conclusion** | | | |
| *Conclusion* | | | |
| **PICO** | | | |
| *Patient* | *Intervention* | *Comparison/Control* | *Outcome* |
| **Search strategy literature databases** | | | |
| *Search engine, search terms, filters, etc.* | | | |
| **Search results literature databases** | | | |
| *Reference* | *Level of evidence* | *Results*<br>*Comments authors study*<br>*Comments pharmacist reviewers* | |
| **Guidelines** | | | |
| *Reference* | | *Results*<br>*Comments pharmacist reviewers* | |
| **Other sources** | | | |
| *Reference other source (e.g. SmPC)* | | *Warning*<br>*Side effects* | |
| **Risk factors** | | | |
| *Identified risk factors* | | *Motivation* | |
| | **Drug-disease interaction** | **Drug alert required** | **Date** |
| **Conclusion expert panel** | *Yes/No* | *Yes/No* | *Date* |
